# Monitoring EPR Effect Dynamics during Nanotaxane Treatment with Theranostic Polymeric Micelles

**DOI:** 10.1002/advs.202103745

**Published:** 2022-01-24

**Authors:** Ilaria Biancacci, Federica De Lorenzi, Benjamin Theek, Xiangyang Bai, Jan‐Niklas May, Lorena Consolino, Maike Baues, Diana Moeckel, Felix Gremse, Saskia von Stillfried, Asmaa El Shafei, Karina Benderski, Armin Azadkhah Shalmani, Alec Wang, Jeffrey Momoh, Quim Peña, Eva Miriam Buhl, Johannes Buyel, Wim Hennink, Fabian Kiessling, Josbert Metselaar, Yang Shi, Twan Lammers

**Affiliations:** ^1^ Department of Nanomedicine and Theranostics Institute for Experimental Molecular Imaging RWTH Aachen University Clinic Aachen 52074 Germany; ^2^ Gremse‐IT GmbH Aachen 52068 Germany; ^3^ Institute of Pathology Medical Faculty RWTH Aachen University Clinic Aachen 52074 Germany; ^4^ Electron Microscopy Facility Institute of Pathology RWTH University Hospital Aachen 52074 Germany; ^5^ Fraunhofer Institute for Molecular Biology and Applied Ecology IME Aachen 52074 Germany; ^6^ Institute of Molecular Biotechnology RWTH Aachen University Aachen 52074 Germany; ^7^ Department of Pharmaceutics Utrecht Institute for Pharmaceutical Sciences Utrecht University Utrecht 3584 CG The Netherlands; ^8^ Institute for Experimental Molecular Imaging RWTH Aachen University Clinic Aachen 52074 Germany; ^9^ Fraunhofer Institute for Medical Image Computing MEVIS Bremen 28359 Germany

**Keywords:** cancer nanomedicine, EPR effect, polymeric micelles, theranostics, tumor targeting

## Abstract

Cancer nanomedicines rely on the enhanced permeability and retention (EPR) effect for efficient target site accumulation. The EPR effect, however, is highly heterogeneous among different tumor types and cancer patients and its extent is expected to dynamically change during the course of nanochemotherapy. Here the authors set out to longitudinally study the dynamics of the EPR effect upon single‐ and double‐dose nanotherapy with fluorophore‐labeled and paclitaxel‐loaded polymeric micelles. Using computed tomography‐fluorescence molecular tomography imaging, it is shown that the extent of nanomedicine tumor accumulation is predictive for therapy outcome. It is also shown that the interindividual heterogeneity in EPR‐based tumor accumulation significantly increases during treatment, especially for more efficient double‐dose nanotaxane therapy. Furthermore, for double‐dose micelle therapy, tumor accumulation significantly increased over time, from 7% injected dose per gram (ID g^–1^) upon the first administration to 15% ID g^–1^ upon the fifth administration, contributing to more efficient inhibition of tumor growth. These findings shed light on the dynamics of the EPR effect during nanomedicine treatment and they exemplify the importance of using imaging in nanomedicine treatment prediction and clinical translation.

## Graphical abstract

Using fluorophore‐labeled and paclitaxel‐loaded polymeric micelles, the authors demonstrate that: 1) the interindividual heterogeneity in nanomedicine tumor accumulation increases during the course of treatment; 2) the extent of nanomedicine tumor accumulation is predictive for therapy outcome; and 3) imaging tumor accumulation multiple times during treatment more favorably predicts therapy outcome than imaging just once at the start of therapy.

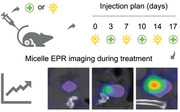

## 1 Introduction

Nanomedicine accumulation in tumors and metastases is often explained on the basis of the Enhanced Permeability and Retention (EPR) effect, first described by Prof. Yasuhiru Matsumara and Prof. Hiroshi Maeda in 1986.^[^ ^1^ ^]^ This widely employed concept for “passive drug targeting” to tumors traditionally relies on the combination of disease‐specific presence of hyperpermeable blood vessels and absence of functional lymphatic drainage.^[^ ^1^ ^]^

Standard chemotherapeutic drugs, with their very small size—typically less than 1 nm—exhibit usually nonspecific distribution in off‐target organs, resulting in adverse effects. Low‐molecular weight drugs also undergo rapid renal clearance and present short circulation half‐lives, severely limiting their accumulation in tumors. Nanomedicine formulations, to the contrary, present reduced renal excretion as well as prolonged circulation times within the blood stream, owing to their dimensions, which are typically above the renal clearance threshold of 7–10 nm.^[^ ^2^ ^]^ This increases the chances of nanomedicine‐based drug delivery systems—and the encapsulated active pharmaceutical ingredients (API) to reach the pathological site, resulting in therapeutically meaningful API concentrations in tumors and metastases, while attenuating API localization in healthy organs and tissues.^[^ ^3^ ^]^

In recent years, several additions/extensions have been made to the list of pathophysiological features contributing to EPR‐mediated passive tumor targeting. The most prominent ones include active transcytosis across the blood vessel wall as a mechanism of nanoparticle extravasation,^[^ ^4^ ^]^ and phagocytic uptake by tumor‐associated macrophages (TAM) as a mechanism of nanoparticle retention.^[^ ^5^ ^]^

From a more chemical and material‐based perspective, substantial progress has also been made over the years with regard to “active tumor targeting”^[^ ^2^, ^6^ ^]^ and a steadily increasing numbers of antibody‐drug conjugates have been approved for clinical use.^[^ ^7^ ^]^ Nevertheless, despite sky‐rocketing numbers of preclinical papers on actively targeted nanomedicines, progress toward successful nanodrug products has been disappointing, with multiple clinical trials not leading to favorable outcomes.^[^ ^2^, ^6^, ^8^ ^]^ On the one hand, this is an integral part of ongoing technology development and the concept and product innovation cycle, which will continuously evolve. On the other hand, 35 years after its conceptualization by Prof. Hiroshi Maeda and Prof. Yasuhiro Matsumura, the EPR effect remains to be one of the main mechanisms contributing to nanomedicine‐based tumor targeting.^[^ ^1^, ^9^ ^]^

Regardless of whether cancer nanomedicines rely only on passive, or on both passive and active tumor targeting, the extent of EPR‐mediated target site localization is crucial to ensure a good treatment outcome.^[^ ^2^, ^3^, ^10^ ^]^ Indeed, by using nanoparticle‐based diagnostic and theranostic agents in combination with noninvasive and quantitative magnetic resonance imaging and positron emission tomography in patients, scientists from Merrimack Pharmaceuticals as well as Mount Sinai/Memorial Sloan together with collaborators demonstrated that a higher degree of target site localization correlates with improved therapeutic responses.^[^ ^11^ ^]^ These findings are based on an imaging measurement after a single dose of diagnostic and theranostic agents, neglecting possible changes in later therapeutic response. This exemplifies the importance of establishing and implementing imaging probes and protocols for patient stratification and clinical translation.

What has remained notoriously elusive over the years is how the tumor accumulation of drugs and drug delivery systems changes during the course of nanochemotherapy. To evaluate this, we employed a model nanoformulation encapsulating a model drug payload, and we longitudinally monitored the extent of nanomedicine tumor accumulation, herein also referred to as EPR effect dynamics, at multiple different time points during treatment. Our theranostic polymeric micelles, based on poly(ethylene glycol) and benzoylated poly(methacrylamide) derivatives, were physically loaded with the clinically extensively used taxane drug, paclitaxel (PTX), via Π‐Π stacking and hydrophobic interactions,^[^ ^12^ ^]^ and chemically labeled in the micelles’ core with the near‐infrared dye Cy7 for noninvasive and quantitative computed tomography–fluorescence molecular tomography (CT‐FMT) imaging.^[^ ^13^ ^]^

**Figure 1:**
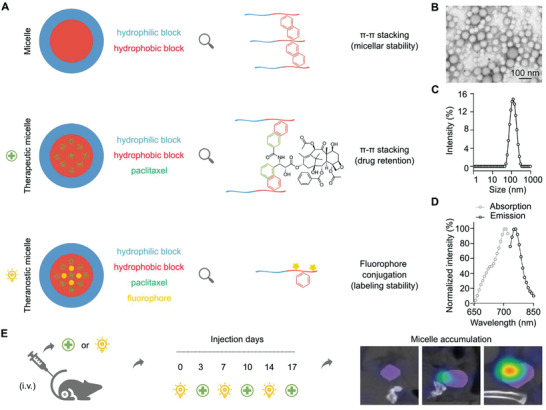
Micelle formulation and study setup. A) Polymeric micelles were physically stabilized by Π‐Π stacking and paclitaxel (PTX) was entrapped in the hydrophobic core with the assistance of Π‐Π stacking to form therapeutic micelles. Cy7 was covalently conjugated in the hydrophobic core of the micelles to provide theranostic micelles. B–D) Characterization of theranostic micelles using transmission electron microscopy (TEM) and fluorometer for their size and absorbance and emission spectra. E) Treatment and imaging protocol: 4T1 tumor‐bearing mice were injected i.v. twice weekly for 3 weeks, alternating therapeutic, and theranostic micelles. Mice were longitudinally monitored via hybrid CT‐FMT imaging on days 0, 3, 7, 10, 14, and 17 to evaluate the dynamics of the EPR effect during the course of nanotaxane therapy.

Well aware of the fact that chemotherapeutics oftentimes display nonlinear dose‐response relationships with steep increases in efficacy even at only moderately increased dosing levels, in mice with orthotopic 4T1 triple‐negative breast cancer (TNBC) tumors, we set out to evaluate the efficacy of theranostic polymeric micelles at two different doses (i.e., 15 and 30 mg kg^–1^ PTX‐equivalent; administered twice weekly), one slightly below and one slightly above the maximum tolerated dose of the clinically approved PTX‐formulation Taxol (20 mg kg^–1^).^[^ ^14^ ^]^ Within this dosing range, the safety profile and therapeutic window of PTX‐based polymeric micelles have been previously established in mice bearing MDA‐MB‐468 TNBC and A431 epidermoid carcinoma tumors.^[^ ^12a^ ^]^ As illustrated in **Figure** **1**, in our study setup, we alternated the injection of single‐ and double‐dose theranostic (on days 0, 7, and 14) and therapeutic micelles (on days 3, 10, and 17), and we systematically studied how (EPR‐based) nanomedicine tumor accumulation correlates with antitumor response, and how the extent of (EPR‐based) nanomedicine tumor accumulation changes during the course of taxane‐based nanochemotherapy.

## 2 Results and Discussion

To visualize and quantify the extent of the EPR effect during nanotaxane treatment, we employed Π electron‐stabilized polymeric micelles with covalently conjugated Cy7 in the core. PTX was physically loaded in the hydrophobic core of the micelles (Figure 1A). The encapsulation efficiency (EE) was about 80% and the loading capacity (LC) around 10%. The micelles were spherically shaped and with a size of around 100 nm (Figure 1B,C). Cy7 was stably conjugated to the micelle‐forming polymers (Figure S1, Supporting Information) and its fluorescence properties were maintained, with absorbance/emission peaks at 764 and 790 nm, respectively (Figure 1D).

**Figure 2:**
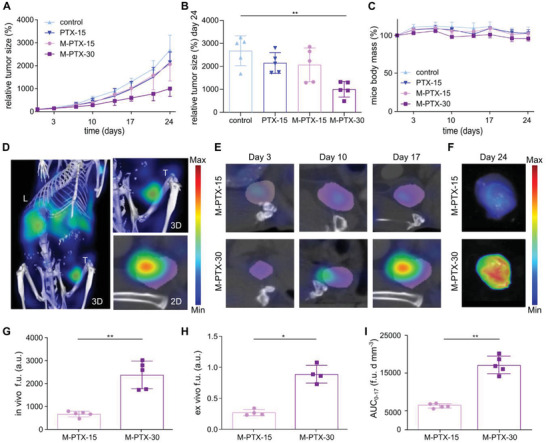
Treatment efficacy and tumor accumulation monitoring of paclitaxel‐loaded polymeric micelles. A,B) At the end of the study, the relative tumor size was significantly decreased for the M‐PTX‐30 group as compared to PBS and free or encapsulated PTX dosed at 15 mg kg^–1^ groups. % values are calculated based on each individual tumor absolute size at day 0. C) Mouse body mass remained constant during treatment, demonstrating the tolerability of the interventions. % values are calculated based on the body mass of each mouse at day 0. D,E) Representative in vivo CT‐FMT images of the tumor localization of theranostic micelles exemplify the stable accumulation pattern for micelles dosed at 15 mg kg^–1^ PTX‐equivalent and the increasing accumulation pattern for micelles dosed at 30 mg kg^–1^ PTX‐equivalent. T = Tumor. L = Liver. F) Ex vivo FRI images of the tumor accumulation of Cy7‐labeled PTX‐micelles at the end of the study, demonstrated higher tumor accumulation for the double‐dosed micelles. G,H) Quantification of the in vivo (CT‐FMT; on day 17) and ex vivo (FRI; on day 24) fluorescence units (f.u.) of Cy7‐labeled PTX‐micelles in tumors showed disproportionally higher accumulation of the double‐dosed micelles after the last micelles injection. I) The cumulative concentrations (AUC; area under the curve in fluorescence units*days*mm^–3^ (f.u. d mm^–3^) of Cy7‐labeled PTX‐micelles in tumors, measured between day 0 and day 17, confirmed the disproportionally higher tumor accumulation for the 30 mg kg^–1^‐dosed micelles versus the 15 mg kg^–1^‐dosed micelles. Values represent average ± SD. * *p* < 0.05, ** *p* < 0.01. Panel B: *n* = 5 per group; unpaired, nonparametric one‐way ANOVA and Dunn's multiple comparison test. Panels G,I: *n* = 5 per group; unpaired, nonparametric, two‐tailed *t*‐test. Panel H: *n* = 4 per group; unpaired, nonparametric, two‐tailed *t*‐test.

Mice bearing orthotopically inoculated 4T1 TNBC were treated twice weekly for 3 weeks (Figure 1E). The mice received either phosphate buffered saline (PBS, control), or free PTX (15 mg kg^–1^), or equivalent and double doses of micellar PTX (M‐PTX; 15 and 30 mg kg^–1^ drug‐equivalent). As compared to PBS, all taxane‐based treatments slowed down tumor growth (**Figure** **2**A). In the aggressively growing and only moderately taxane‐responsive 4T1 model, a statistically significant reduction in tumor growth was only observed for micellar PTX administered at a dose of 30 mg kg^–1^ (Figure 2B; *p* < 0.01, based analysis of variance (ANOVA), using an alpha threshold of 0.05).^[^ ^15^ ^]^ These findings were validated by demonstrating decreased proliferation and increased tissue damage in tumor sections upon treatment with the highest dose of micellar PTX (Figures S2 and S3, Supporting Information). All treatments were found to be well tolerated, as evidenced by stable mouse body mass, as well as general good condition and behavior of the mice throughout the course of the therapy (Figure 2C).

**Figure 3:**
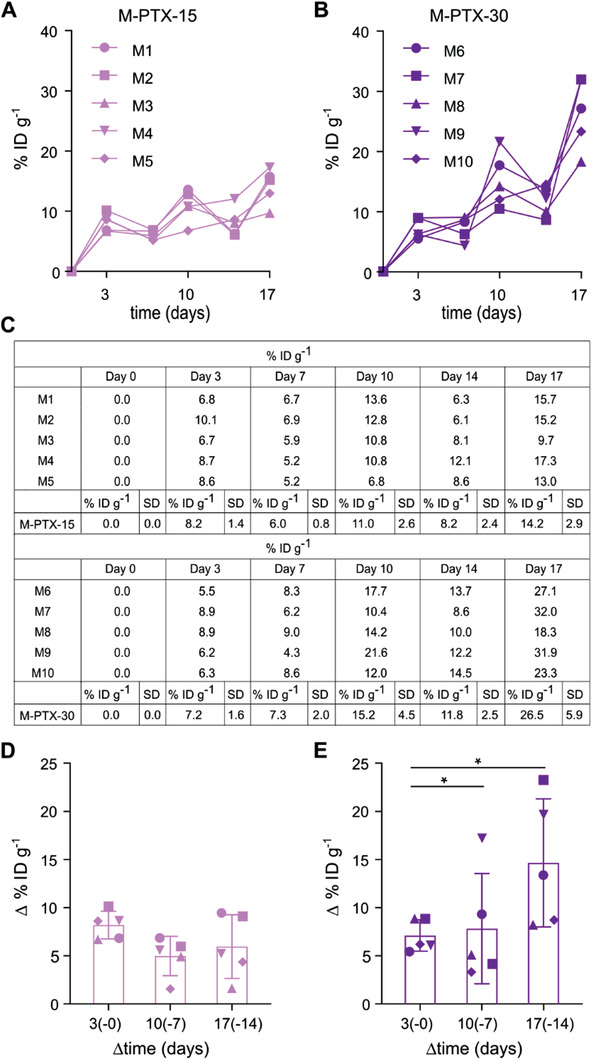
Quantitative assessment of individual EPR effect dynamics during treatment. A,B) Theranostic micelles were administered on days 0, 7, and 14, and therapeutic micelles on days 3, 10, and 17. Comparable % ID g^–1^ of micelles were found in 4T1 tumors after the 1st injection for the 15 and the 30 mg kg^–1^ groups, while cumulative accumulation patterns upon subsequent injections indicated a disproportionally large increase in EPR‐mediated tumor accumulation for the 30 mg kg^–1^ group. C) The table summarizes the % ID g^–1^ values for the 15 and 30 mg kg^–1^ groups on days 0, 3, 7, 10, 14 and 17. D,E) The evolution of and variability in EPR‐mediated micelle accumulation in tumors was quantified via determining the difference (i.e., ∆ % ID g^–1^) between the % ID g^–1^ of Cy7‐PTX‐micelles accumulated 3 d after i.v. injection and the % ID g^–1^ of Cy7‐PTX‐micelles present in tumors just before that respective i.v. injection. The ∆ % ID g^–1^ values slightly decreased on average over time for the 15 mg kg^–1^ group and slightly increased for the 30 mg kg^–1^ group. Inter‐individual variability in EPR‐mediated tumor accumulation increased for both groups. Values represent average ± SD (*n* = 5 per group). *F*‐tests on unpaired, parametric, two‐tailed t tests with Welch's correction were performed to compare variance between the different time points. In addition, paired, parametric, two‐tailed *t*‐tests were performed to compare ∆ % ID g^–1^ values between each injection for both groups. Unpaired, parametric, two‐tailed *t*‐tests were performed to assess differences in micelle accumulation (∆ % ID g^–1^) between M‐PTX‐15 and M‐PTX‐30 at the different time points. * *p *< 0.05.

To longitudinally assess the tumor accumulation of the PTX‐micelles (M‐PTX) during treatment, the 1st, 3rd, and 5th injection were done with Cy7‐labeled micelles. Hybrid CT‐FMT imaging was always performed previous and 72 h after theranostic micelles administration, right before the administration of the second weekly dose of the therapeutic micelles (i.e., non‐Cy7‐containing PTX‐micelles; 2nd, 4th, and 6th injection; Figure 1E). Such study design allowed for the dynamic monitoring of polymeric micelles accumulation and retention at malignant sites as well as for the monitoring of the therapy‐induced variability therein. At the same time, via alternating theranostic and therapeutic micelles, we maintained the regimen of twice weekly therapy without having an interference of overlapped fluorescence signals between subsequent injections. As shown in Figure 2D,E and Figure S4 in the Supporting Information, the whole‐body biodistribution, tumor accumulation and healthy tissue localization of theranostic micelles were visualized at day 3, day 10, and day 17 after the start of therapy. On day 3, both the low and the high dose of theranostic micelles showed a similar tumor accumulation pattern, with nearly identical relative values for tumor concentration, normalized to the injected dose and expressed as a percentage of the injected dose per gram tumor tissue (% ID g^–1^). For the 15 mg kg^–1^ PTX‐micelles group, 8.2 ± 1.4% ID g^–1^ was achieved, versus 7.2 ± 1.6% ID g^–1^ for the 30 mg kg^–1^ group (**Figure** **3**A–C). This shows that for the polymeric micelles, at this initial time point of therapy and within this relatively limited dosing range (i.e., factor 2), there is no dose‐dependent effect on EPR‐mediated tumor accumulation.^[^ ^16^ ^]^

Interestingly, beyond the first week of therapy, dose‐dependent differences in polymeric micelle tumor targeting started to become apparent between the two treatment groups. The double‐dosed micelles (M‐PTX‐30) showed a steady increase in tumor accumulation over time, whereas for the single‐dosed micelles (M‐PTX‐15), no gradual increase was observed (see Figure 2E). Ex vivo fluorescence reflectance imaging (FRI) of resected tumors at the end of therapy confirmed the overall superior tumor accumulation for micelles dosed at 30 mg kg^–1^ (Figure 2F). Quantification of tumor fluorescence after the last administration of theranostic micelles verified the higher accumulation of the 30 mg kg^–1^‐dosed micelles as compared to the 15 mg kg^–1^‐dosed micelles, both in vivo (2376 vs 673 fluorescence units (f.u.); Figure 2G) and ex vivo (0.9 vs 0.3 f.u.; Figure 2H and Figure S5, Supporting Information). In line with this, analysis of fluorescence signals in 100 µm thick tumor sections showed a more favorable intratumoral distribution upon treatment with the double dose, as evidenced by deeper penetration of the micelles into the core of the tumor (Figure S5C–F, Supporting Information). Subsequent microscopy analysis of Cy7‐micelle distribution showed a threefold increase in the accumulation of micelles for the M‐PTX‐30 group, and it confirmed higher levels of micelle accumulation in the inner part of the tumors, albeit with higher levels of intertumor variability as compared to the M‐PTX‐15 group (Figure S6, Supporting Information).

Longitudinal CT‐FMT imaging corroborated these results via analyzing the total tumor accumulation over time, i.e., the area under the curve (AUC), which was also found to be disproportionally higher for the M‐PTX‐30 group than for the M‐PTX‐15 group (6599 vs 17 087 fluorescence units*days*mm^–3^ (f.u. d mm^–3^); *p* < 0.01; Figure 2I). This indicates that efficient cancer nanotherapy positively promotes the extent of (EPR‐based) nanomedicine tumor accumulation, and vice versa, that improved (EPR‐based) nanomedicine tumor targeting seems to be able to positively influence treatment outcomes. We are fully aware of the fact that these results cannot yet be generalized, because they only reflect results obtained in a single tumor model, responding to a single drug (PTX), loaded in a single nanomedicine formulation (poly(ethylene glycol)‐benzoylated poly(methacrylamide) (PEG‐PHPMA‐Bz)‐based micelles) and tested in a limited dosing range (15–30 mg kg^−1^). Moreover, it is unclear to which extent pure EPR‐based features such as enhanced vascular leakiness and constrained lymphatic drainage contribute to these treatment‐induced changes, as compared to, e.g., active endothelial transcytosis, macrophage‐mediated retention and other treatment‐induced (patho)physiological effects affecting nanomedicine tumor accumulation. We nonetheless believe that these initial longitudinal imaging and conceptual patient stratification efforts are valuable for helping to shape rational ways forward toward an improved understanding and more efficient translation of cancer nanomedicine therapies.

Extensive immunofluorescence and histopathological microscopy analyses, as well as ex vivo micro‐CT imaging, were conducted to investigate the effect of taxane‐based (nano)therapy on features of the tumor vascularization and the microenvironment. In good agreement with the disproportionally enhanced tumor accumulation results reported above, it was found that treatment with M‐PTX‐30 beneficially affected the tumor vascularization, as evidenced by a higher degree of blood vessel functionality and maturation (Figures S7 and S8, Supporting Information). Micellar nanotherapy furthermore resulted in a decrease in tumor cell density, in tumor‐associated macrophage density and in collagen content, again in a dose‐dependent manner (Figures S9 and S10, Supporting Information). Altogether, this points toward a positive feedback loop between dose‐ and treatment‐mediated modulation of the tumor microenvironment on the one hand and better nanomedicine accumulation and efficacy on the other hand.

With regard to dose‐ and therapy‐dependent differences in micelle tumor accumulation over time, similar relative % ID g^–1^ of micelles localized to tumors at day 3 for both dosing groups. These % ID g^–1^ remained comparable until day 7, with 6.0 ± 0.8% ID g^–1^ for the 15 mg kg^–1^ group and 7.3 ± 2.0% ID g^–1^ for the 30 mg kg^–1^ group (Figure 3A–C). The first noticeable differences in EPR‐mediated tumor accumulation and retention started to become apparent between the M‐PTX‐15 and the M‐PTX‐30 group on days 10 and 14, after several PTX‐micelles injections. Upon the 3rd injection of PTX‐micelles, which took place immediately after the CT‐FMT scan on day 7, the % ID g^–1^, calculated on the basis of imaging measurements, revealed values of 11.0 ± 2.6 versus 15.2 ± 4.5 at day 10, and 8.2 ± 2.4 versus 11.8 ± 2.5 at day 14, respectively (Figure 3A–C). This discrepancy was more pronounced after the 5th injection of PTX‐micelles, which was administered on day 14. Three days later, on day 17, a tumor accumulation of 14.2 ± 2.9% ID g^–1^ was observed for the 15 mg kg^–1^ group, as compared to 26.5 ± 5.9% ID g^–1^ for the 30 mg kg^–1^ group (Figure 3A–C). It again needs to be stressed that these values are normalized to the injected dose (i.e., expressed as % ID g^–1^), and that similar % ID g^–1^ values would thus be expected if nanodrug treatment would not affect the extent of the EPR effect. The observed 187% increase in relative tumor accumulation for the 30 mg kg^–1^ versus the 15 mg kg^–1^ group at day 17 therefore suggests a positive feedback loop between successful antitumor nanochemotherapy and enhanced nanomedicine accumulation.

To better understand the dynamics of (EPR‐based) nanomedicine tumor accumulation over time and during treatment, we next quantified the % ID g^–1^ of micelles delivered to tumors at each individual injection. This was done by subtracting the residual % ID g^–1^ of theranostic polymeric micelles in tumors just prior to the next injection, from the % ID g^–1^ of micelles present in tumors 3 d after the respective injection. As an example, this refers to the difference in tumor accumulation on day 10 (i.e., the % ID g^–1^ at 3 d after the 2nd injection of theranostic micelles) and day 7 (i.e., the % ID g^–1^ just before the 2nd injection of theranostic micelles). We refer to this as ∆ % ID g^–1^ (Figure 3D,E). For the 15 mg kg^–1^ PTX‐micelles group, the values of ∆ % ID g^–1^ slightly decreased over time, from 8.2% ID g^–1^ after the 1st injection, to 5.0% ID g^–1^ after the 2nd injection, and to 6.0% ID g^–1^ after the 3rd injection. Conversely, in line with the findings alluded to above, a progressive increase in EPR‐mediated tumor accumulation was observed for micelles dosed at 30 mg kg^–1^ PTX‐equivalent: 7.2% ID g^–1^ accumulated in tumors after the 1st injection, versus 7.9% ID g^–1^ after the 2nd injection, and 14.7% ID g^–1^ after the 3rd injection. Importantly, the tumor accumulation after the 3rd injection was found to be significantly and disproportionally higher for the double‐dosed micelles as compared to the single‐dosed micelles (*p* < 0.05) (Figure 3D,E).

A second and arguably even more important observation that becomes clear upon analyzing the data (see Figure 3) is that the heterogeneity in EPR‐mediated micelle accumulation between the tumors increased over time and with treatment. Both for the 15 mg kg^–1^ and for the 30 mg kg^–1^ group, considerably more interindividual variability is noticed going up from day 3 to day 10 to day 17. This holds true both for the cumulative tumor accumulation analyses in Figure 3A,B as well as for the ∆ % ID g^–1^ values in Figure 3D,E. To exemplify this, at days 3, 10, and 17 during therapy follow‐up, the standard deviation (SD) increases from 1.4 to 2.0 and to 3.3 for the 15 mg kg^–1^ group, and from 1.6 to 5.7 and to 6.7 for the 30 mg kg^–1^ group. For the double‐dose group, *F*‐test‐based analysis of variability produces statistically significant *p*‐values for the comparison of day 3 versus day 10 (*p* = 0.03), and for day 3 versus day 17 (*p* = 0.02). This indicates that in the case of successful nanodrug therapy, variability in EPR‐mediated nanomedicine accumulation in tumors increases, thus influencing the response to the therapy. This holds potential for patient stratification and future translation to the clinic.

**Figure 4:**
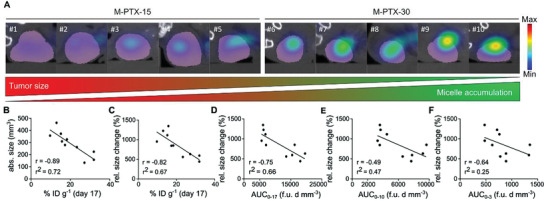
Nanomedicine tumor accumulation correlates with antitumor response. A) 2D CT‐FMT images on the transverse plane taken on day 17 show that tumors which strongly accumulate PTX‐micelles have smaller sizes than tumors which accumulate micelles less efficiently. Tumor accumulation is shown as blue‐to‐red fluorescence clouds. CT‐segmentations of tumors are presented in purple. B–F) Correlation of static and dynamic polymeric micelle tumor accumulation with therapy outcome. Static assessment of polymeric micelle accumulation at day 17 (i.e., after three theranostic and three therapeutic doses) shows a very good correlation with antitumor response for both B) absolute and C) relative tumor growth kinetics. Dynamic assessment of cumulative micelle accumulation over time (i.e., the area under the curve; AUC) also correlated well with therapy outcome. The relative size change in tumor growth (in %) between day 3 and 24 was correlated with micelle AUC (fluorescence units*days*mm^–3^ (f.u. d mm^–3^)) upon D) all three, E) the first two, and F) only the first injection, showing that prolonged monitoring produces more favorable results. Spearman´s correlation coefficient (*r*) and goodness‐of‐fit for linear regression (*r* ^2^) using alpha threshold of 0.05 were calculated using GraphPadPrism 9.

We ultimately evaluated how noninvasive imaging of polymeric micelles tumor accumulation correlates with (and might eventually be employed to predict)^[^ ^9^ ^]^ cancer nanomedicine treatment efficacy. This was done both dynamically (i.e., correlating tumor growth as relative size change between day 3 and day 24, and tumor accumulation as AUC between day 0 and 17), and statically (i.e., correlating tumor size at day 24 (last day of treatment follow‐up) with micelle tumor accumulation at day 17 (last day of imaging)), aiming to explore the added value of prolonged longitudinal imaging during the course of therapy versus imaging just once on (or just before) the first day of treatment. As exemplified by the CT‐FMT images in **Figure** **4**A, which were obtained at the last imaging time point at day 17, we observed a very good correlation between the extent of polymeric micelles tumor accumulation and the efficacy of micelle‐based nanochemotherapy. This notion is validated via extensive immunofluorescence and histopathological analyses (Figures S2 and S3, Supporting Information).

As shown in Figure 4B, when correlating the tumor size with micelle tumor accumulation, a significant (*p* value < 0.01) Spearman's correlation coefficient (r) of 0.89 was obtained, with a goodness‐of‐fit (*r* ^2^) of 0.72. Tumor growth over time, i.e., the relative size change in % between day 3 and day 24, also significantly correlated (*p* < 0.01) with micelle tumor accumulation at day 17, with *r* = 0.82 and *r* ^2^ = 0.67 (Figure 4C). When correlating antitumor response with tumor accumulation over time, the correlation was similarly reliable (*p* < 0.05), with a Spearman's correlation coefficient of 0.72 and a goodness‐of‐fit of 0.66 (Figure 4D). Reducing the dynamic imaging time‐window from day 0–17 to day 0–10 and to day 0–3 substantially reduced the correlation between micelle accumulation and antitumor response, with Spearman's correlation values of 0.49 and 0.64, and goodness‐of‐fit ‐values of 0.47 and 0.25, respectively (Figure 4E,F). Furthermore, the statistical significance of the correlations decreased, with *p* values > 0.05 (no‐significant) both for the intermediate (0–10), and for the short (0–3) imaging time‐frames (Figure 4E,F). As expected, imaging micelle tumor accumulation regularly over time is a more suitable predictor of therapy outcome than imaging just once or twice at the beginning of the treatment. This likely results from gradual changes in the extent of the EPR effect during treatment, which cannot be properly captured and quantified when imaging tumor accumulation only once or twice early on in the course of therapy.

## 3 Conclusion

Cy7‐labeled and PTX‐loaded polymeric micelles were generated to longitudinally visualize and quantify nanomedicine tumor accumulation during nanotaxane therapy of 4T1 murine triple‐negative breast cancer tumors. Our findings show a dose‐ and treatment efficacy‐dependent accumulation of micelles in tumors. Heterogeneity in EPR‐based tumor targeting is for the first time shown to increase during nanomedicine therapy. The disproportionally higher tumor accumulation observed for double‐dosed micelles enforced and better predicted therapy outcome. Imaging micelle tumor accumulation multiple times during treatment more favorably predicted therapy outcomes than imaging just once at the beginning of therapy. Together, these findings showcase the importance of involving noninvasive and quantitative imaging in nanomedicine treatment prediction and clinical translation

## 4 Experimental Section

### Preparation and Characterization of Polymeric Micelles

Polymeric micelles were formulated using block copolymers based on poly(ethylene glycol) and benzoylated poly(methacrylamide) derivatives and covalently core‐labeled with the near‐infrared dye Cy7 as described in ref. [12a]. Information on the labeling protocol and the stability of dye conjugation is the Supporting Information. Micelles were prepared using the nanoprecipitation method. In brief, 27 mg of the polymer and 4.5 mg of PTX were dissolved in 1 mL of tetrahydrofuran (THF) and the solution was added dropwise to 1 mL of deionized ultrafiltered water under stirring at room temperature (RT). The mixture was kept at RT for 48 h to evaporate THF. The resulting formulation was filtered with nylon membranes (pore size: 0.45 µm) and stored at RT prior to in vitro and in vivo evaluations. The size of the polymeric micelles was determined by dynamic light scattering (Nano‐s, Malvern Instruments Ltd., UK) and transmission electron microscopy (TEM; Leo 906, Zeiss, Germany). PTX concentration was determined by high performance liquid chromatography (LC‐20AT, Shimadzu, Japan). EE and LC were calculated as follows

(1) $$\;\mathrm{EE}\%=\;\frac{\text{Detected}\;\text{amount}\;\text{of}\;\text{drug}}{\text{Feed}\;\text{amount}\;\text{of}\;\text{drug}}\times 100$$

(2) $$\mathrm{LC}=\;\frac{\text{Detected}\;\text{amount}\;\text{of}\;\text{drug}}{\text{Detected}\;\text{amount}\;\text{of}\;\text{drug}\;+\;\text{feed}\;\text{amount}\;\text{of}\;\text{polymer}}\times 100$$

### Therapy Study

All animal experiments performed at the Institute for Experimental Molecular Imaging at Rheinisch‐Westfälische Technische Hochschule (RWTH) Aachen University Clinic were performed in accordance with the Guide for the Care and Use of Laboratory Animals and were approved by governmental animal welfare authorities (Az. 87‐51.04.2010.A278). Twenty female CD‐1‐Foxn1 nude mice (6–8 weeks old, Charles River) were kept in pathogen‐free cages under a light/dark cycle of 12/12 h and with separate ventilation, food and water were given ad‐libitum. 4T1 TNBC cells (American Type Culture Collection, Manassas, VA, USA) were cultured in Roswell Park Memorial Institute (RPMI) medium (RPMI 1640; Gibco, Life Technologies GmbH, Germany), supplemented with 10% fetal bovine serum (Life Technologies GmbH, Germany) and 1% penicillin/streptomycin (10000 U mL^–1^ penicillin; 10 mg mL^–1^ streptomycin, Life Technologies GmbH, Germany). Mice were orthotopically inoculated with 5*10^5^ 4T1 cells and tumor growth was monitored daily via caliper measurements. When tumors reached a size of 5–6 mm in diameter, mice were randomly divided into four groups, with five mice each group. Anesthesia was applied using 4% isoflurane (Forene, Abbott, Wiesbaden, Germany) in oxygen‐enriched air using a dedicated vaporizer. During scan acquisitions, the isoflurane concentration was reduced to 2.0% and the eyes were kept hydrated with Bepanthen eye ointment (Bayer Vital GmbH, Germany). All treatments were intravenously injected into the lateral tail vein of the mice using a catheter consisting of a 30 G cannula (B.Braun, Melsungen, Germany) and a polyethylene tube (Hartenstein, Würzburg, Germany). Two control groups were included, the first one administered with PBS (Life Technology, Germany) and the second one treated with the commercially available Taxol (dosed at 15 mg kg^–1^). The other two groups were administered with PTX‐loaded micelles either at an equi‐dose (15 mg kg^–1^) or at a double‐dose (30 mg kg^–1^) as compared to Taxol. Treatments were administered twice weekly and micelles were alternatively injected as Cy7‐labeled and nonlabeled.

### Imaging Protocol

Anesthetized mice were subjected to CT measurements in combination with FMT (PerkinElmer, USA). Scans were acquired prior to the injection of fluorescent micelles to obtain a background representation of the previous administration of micellar nanomedicines in the tumor. Three days after injection, scans were acquired and the actual micelle tumor accumulation was assessed by subtracting the background fluorescence signal from the newly obtained fluorescence information. Cy7‐labeled theranostic micelles were i.v. administered on days 0, 7, and 14 and analyzed via CT‐FMT on days 3, 10, and 17. Purely therapeutic micelles were administered in between, just after imaging, on days 3, 10, and 17. Tumor sizes were evaluated twice weekly until day 24 via caliper measurements and CT assessment. On day 24, prior to sacrifice, mice received i.v. injection of fluorescein isothiocyanate (FITC) lectin in order to identify functional and perfused vessels. Tumors were resected from the mice and 2D FRI were acquired. The fluorescence scale was adjusted in a range of 0–0.5 for fair comparison between all tumors. Imalytics Preclinical 2.0 (Gremse‐IT, Germany) was employed to reconstruct, fuse, and analyze the CT and FMT data sets.^[^ ^17^ ^]^

### Statistical Analysis

The intensity of absorbance and emission wavelengths as well as the intensity of the retention time of free Cy7 and conjugated Cy7 were normalized considering the maximum value as 100%. All other values are presented as average ± SD of the actual measurements. Statistical analyses were performed using GraphPadPrism 9 (GraphPad Software, USA). In all analyses, 0.05 was used as alpha threshold for determining statistical significances. Significant differences were considered for *p* values < 0.05 (*), *p* < 0.01 (**), and *p* < 0.001 (***).

Tumor sizes were normalized considering the absolute size of each tumor on day 0 of therapy as 100 to obtain relative tumor sizes which were plotted as average (sample size *n* = 5 per group) ± SD and compared via unpaired, nonparametric one‐way ANOVA analysis with Dunn's multiple comparison test. Unpaired, nonparametric, two‐tailed *t*‐tests were applied to compare the FMT‐based in vivo (*n* = 5 per group ± SD) and ex vivo (*n* = 4 per group ± SD) fluorescence intensity, as well as the final cumulative concentration of the Cy7‐labeled PTX‐micelles in 15 mg kg^–1^ versus 30 mg kg^–1^ groups (*n* = 5 per group ± SD). Paired, parametric, two‐tailed *t*‐tests were performed to compare the micelle accumulation between each injection (∆ % ID g^–1^) of 15 and 30 mg kg^–1^‐dosed micelles (*n* = 5 per group ± SD). Furthermore, *F*‐tests on unpaired, parametric, two‐tailed t tests with Welch's correction were applied to analyze the variability of the micelle accumulation in different tumors after each injection. In addition, unpaired, parametric, two‐tailed *t*‐tests were performed to compare ∆ % ID g^–1^ between 15 and 30 mg kg^–1^‐dosed micelles at each time point as well as the area fraction of the micelles in the core versus periphery, and to compare the amount of intravascular versus extravasated micelles. Goodness of fit (*r* ^2^) of linear regression and Spearman´s correlation coefficient (*r*), using alpha threshold of 0.05 for testing significance of correlations, were calculated for static and dynamic correlations between tumor size/size change (ratio between tumor size on day 24 and on day 3 multiplied by 100) and tumor accumulation (AUC between day 0 and 3, 0 and 10, 0 and 17, *n* = 10 individuals).

For fluorescence stainings and H&E analysis, data are presented as average ± SD (*n* = 4 per group; note that one mouse from all groups was used for ex vivo CT scans of vessel perfusion, and thus not included in ex vivo microscopy analyses). Statistical differences for Ki67, H&E, lectin #, CD31 #, *α*SMA/CD31, 4′,6‐diamidino‐2‐phenylindole (DAPI) #, DAPI area fraction (AF), F4/80 #, CD206 #, and Col I were analyzed via unpaired, parametric one‐way ANOVA with Tukey correction. Unpaired, nonparametric one‐way ANOVA with Dunn's correction was performed to analyze statistical differences for Casp‐3 and lectin/CD31. Data of vasculature features were further split in core versus periphery (*n* = 4 for each group). Statistical differences between core and periphery were analyzed via two‐way ANOVA with Bonferroni correction. For in vivo biodistribution analysis, data are presented as average (*n* = 5 per group) ± SD. Statistical significance between different time points within each organ was analyzed via two‐way ANOVA with Tukey correction.

## Conflict of Interest

The authors declare no conflict of interest.

## Supporting information

Supporting Information

## Data Availability

The data that support the findings of this study are available from the corresponding author upon reasonable request.
