# Supplementary material for: Monitoring EPR Effect Dynamics during Nanotaxane Treatment with Theranostic Polymeric Micelles
Source: Adv Sci (Weinh). 2022 Jan 24;9(10):2103745. doi: 10.1002/advs.202103745 (PMC8981450; doi:10.1002/advs.202103745)
Supplement: Supplementary file 1 — Supporting Information [file ADVS-9-2103745-s001.pdf]

ADVANCED SCIENCE
Open Access

Supporting Information

for *Adv. Sci.*, DOI 10.1002/advs.202103745

Monitoring EPR Effect Dynamics during Nanotaxane Treatment with Theranostic Polymeric Micelles

Ilaria Biancacci, Federica De Lorenzi, Benjamin Theek, Xiangyang Bai, Jan-Niklas May, Lorena Consolino, Maike Baues, Diana Moeckel, Felix Gremse, Saskia von Stillfried, Asmaa El Shafei, Karina Benderski, Armin Azadkhah Shalmani, Alec Wang, Jeffrey Momoh, Quim Peña, Eva Miriam Buhl, Johannes Buyel, Wim Hennink, Fabian Kiessling, Josbert Metselaar, Yang Shi and Twan Lammers**

ADVANCED SCIENCE Open Access

Supporting Information

for *Adv. Sci.,* DOI: 10.1002/advs.202103745

Monitoring EPR Effect Dynamics during Nano-Taxane Treatment with Theranostic Polymeric Micelles

Ilaria Biancacci[1,], Federica De Lorenzi[1,*], Benjamin Theek[1,*], Xiangyang Bai[1], Jan-Niklas May[1], Lorena Consolino[1], Maike Baues[1], Diana Moeckel[1], Felix Gremse[1,2], Saskia von Stillfried[3], Asmaa El Shafei[1], Karina Benderski[1], Armin Azadkhah Shalmani[1], Alec Wang[1], Jeffrey Momoh[1], Quim Peña[1], Eva Miriam Buhl[4], Johannes Buyel[5,6], Wim Hennink[7], Fabian Kiessling[8,9], Josbert Metselaar[1], Yang Shi[1,#], Twan Lammers[1,#]*

# - SUPPORTING INFORMATION -

Monitoring EPR Effect Dynamics during Nano-Taxane Treatment with Theranostic Polymeric Micelles

Ilaria Biancacci[1,*], Federica De Lorenzi[1,*], Benjamin Theek[1,*], Xiangyang Bai[1], Jan-Niklas May[1], Lorena Consolino[1], Maike Baues[1], Diana Moeckel[1], Felix Gremse[1,2], Saskia von Stillfried[3], Asmaa El Shafei[1], Karina Benderski[1], Armin Azadkhah Shalmani[1], Alec Wang[1], Jeffrey Momoh[1], Quim Peña[1], Eva Miriam Buhl[4], Johannes Buyel[5,6], Wim Hennink[7], Fabian Kiessling[8,9], Josbert Metselaar[1], Yang Shi[1,#], Twan Lammers[1,#]

1: Department of Nanomedicine and Theranostics, Institute for Experimental Molecular Imaging, RWTH Aachen University Clinic, Aachen, Germany
2: Gremse-IT GmbH, Aachen, Germany
3: Institute of Pathology, Medical Faculty, RWTH Aachen University Clinic, Aachen, Germany
4: Electron Microscopy Facility, Institute of Pathology, RWTH University Hospital, Aachen, Germany
5: Fraunhofer Institute for Molecular Biology and Applied Ecology IME, Aachen, Germany
6: Institute of Molecular Biotechnology, RWTH Aachen University
7: Department of Pharmaceutics, Utrecht Institute for Pharmaceutical Sciences, Utrecht University, Utrecht, the Netherlands
8: Institute for Experimental Molecular Imaging, RWTH Aachen University Clinic, Aachen, Germany
9: Fraunhofer Institute for Medical Image Computing MEVIS, Bremen, Germany

* Equal contribution

[#] Correspondence: yshi@ukaachen.de, tlammers@ukaachen.de

SUPPORTING FIGURES

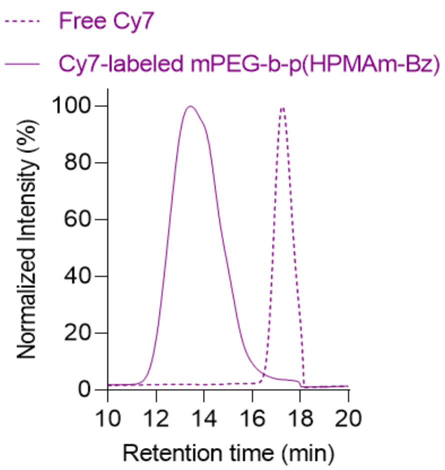

Figure S1: GPC chromatograms of free Cy7 and Cy7-labeled mPEG-b-p(HPMAm-Bz) after purification by dialysis, confirming stable dye conjugation.

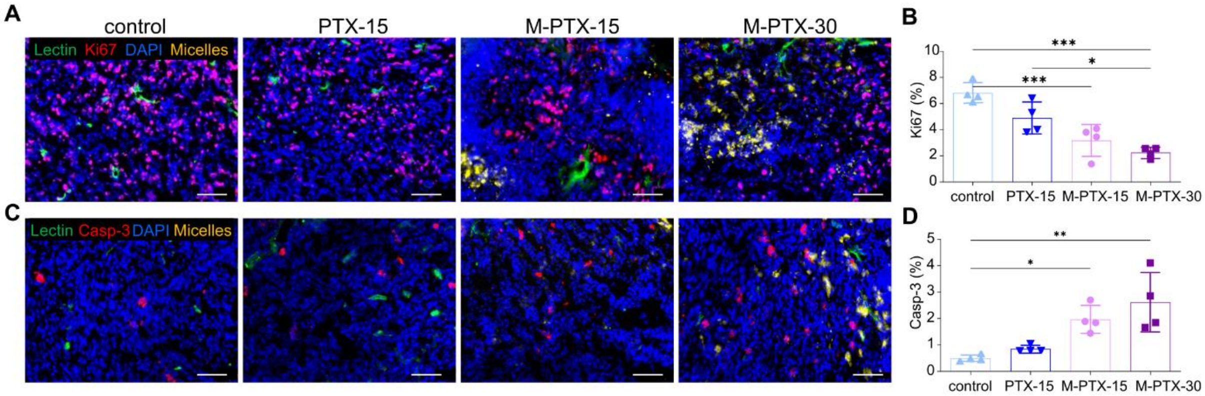

Figure S2: Validation of (nano)therapy responses via immunofluorescence microscopy. A: Representative fluorescence microscopy images showing Ki67$^+$ (red) proliferating cells in control, PTX-15, M-PTX-15 and M-PTX-30 groups. **B**: Image analysis confirmed the improved therapeutic effect of the micelles as compared to the free drug. Significantly reduced Ki67 signal was found for both micelles groups as compared to the control group, but not for free drug. In addition, tumors in mice treated with double-dose micelles showed significantly less proliferation than free PTX. Data are presented as average ± SD (n = 4 per group). Statistical differences were analyzed via unpaired, parametric one-way ANOVA with Tukey correction. P values: * < 0.05, *** < 0.001. C: Representative fluorescence microscopy images showing Caspase-3$^+$ (Casp-3, red) apoptotic cells in control, PTX-15, M-PTX-15 and M-PTX-30 groups. Scale bar = 60 µm. D: Treatment with micellar PTX (but not free PTX) induced a significantly higher degree of apoptosis as compared to the control group. Data are presented as average ± SD (n = 4 per group). Statistical differences were analyzed via unpaired, non-parametric one-way ANOVA with Dunn's correction. P values: * < 0.05, ** < 0.01.

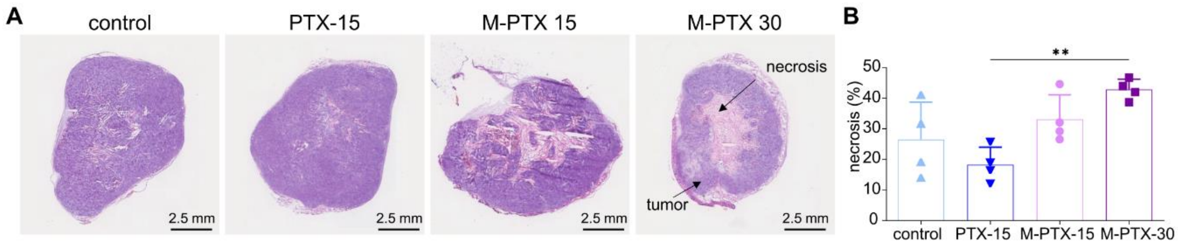

Figure S3: Histopathological analysis of tumor tissue damage. A: Hematoxylin and eosin (H&E) staining showing necrotic areas in all groups. **B**: The extent of necrosis was quantified as the ratio (in %) of the segmented necrotic area and the total area of the tumor. Significantly more necrosis was observed for the M-PTX-30 group as compared to the free drug group. Data are presented as average ± SD (n = 4 per group). Unpaired, parametric one-way ANOVA with Tukey correction was performed to compare the necrotic areas between the different groups. P values: ** < 0.01.

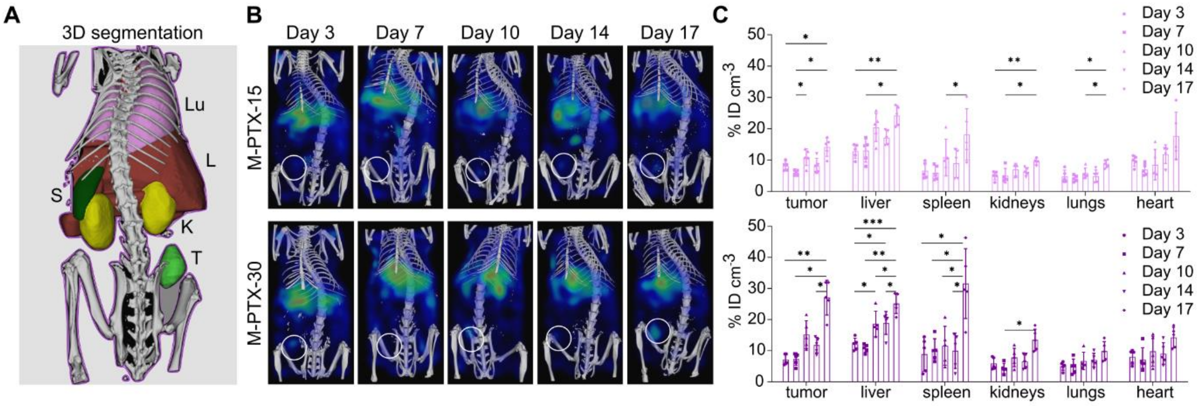

Figure S4: In vivo biodistribution analysis. A: 3D reconstruction of organ segmentation in CT scans. Lu = lungs, L = liver, S = spleen, K = kidneys, T = tumor. **B**: Biodistribution of Cy7-labeled micelles in the M-PTX-15 and M-PTX-30 therapy groups assessed via CT-FMT. **C**: Quantification of micelle biodistribution and target site localization demonstrates a higher tumor accumulation of M-PTX-30 over time. Comparable healthy tissues levels were observed for all healthy organs apart from spleen on day 17 (which was also higher for M-PTX-30). Data are presented as average ± SD (n = 5 per group). Statistical significance between different time points within each organ was analyzed via two-way ANOVA with Tukey correction. P values: * < 0.05, ** < 0.01, *** < 0.001.

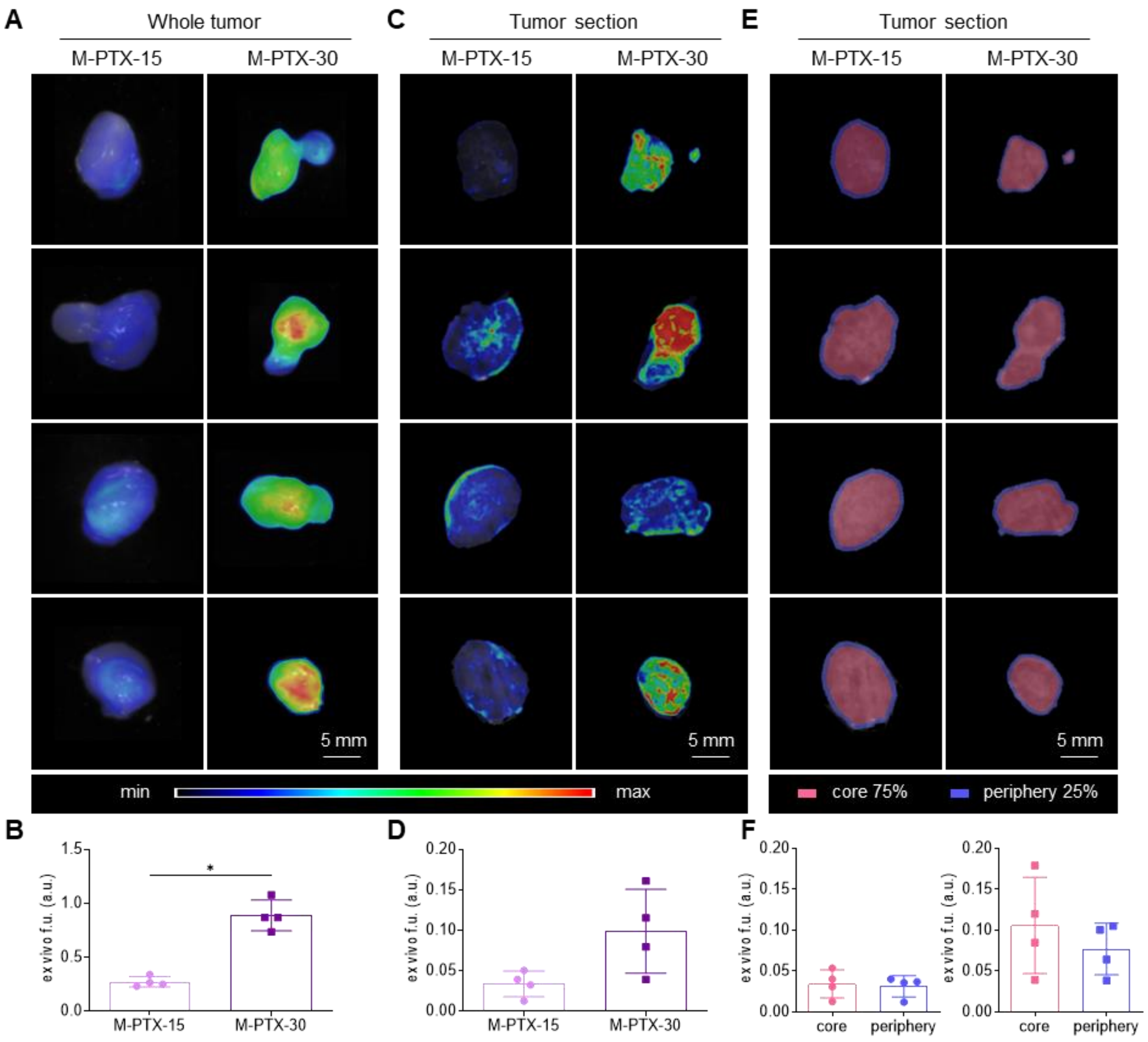

Figure S5: Macroscopic ex vivo analysis of micelle accumulation and distribution. A-B: Quantification of ex vivo fluorescence units (f.u.) of Cy7-labeled PTX-micelles in tumors showed significantly higher accumulation of the double-dosed micelles. **C-D**: Fluorescence was also quantified in 100 µm-thick tumor sections. Higher accumulation and higher inter-tumor variability was observed in the M-PTX-30 group as compared to the M-PTX-15 group. **E-F**: Cy7-labeled micelles showed a more favorable tumor distribution pattern upon treatment with the double dose, as evidenced by the relatively deeper penetration of the micelles into the core of the tumor. Data are presented as average ± SD (n = 5 per group). Unpaired, non-parametric two-tailed t-tests were performed to compare the ex vivo fluorescence images. * < 0.05.

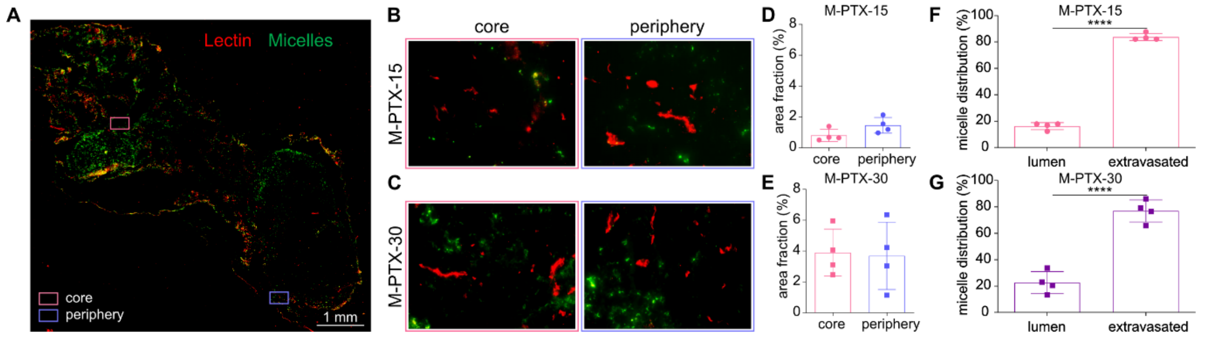

Figure S6: Microscopic ex vivo analysis of micelle accumulation and distribution. A: Representative whole-tumor fluorescence microscopy image showing perfused blood vessels (lectin) and micelles in tumor tissue. As in Figure S5, tumors were divided into two regions, considering the inner 75% as the core and the outer 25% as the periphery. **B-C**: Representative zoom-in images of tumor core and periphery for the single- and double-dose treatment groups, exemplifying the accumulation and distribution patterns of the micelles. **D-E**: Microscopically, micelles in the M-PTX-15 group accumulated preferentially in the periphery. Conversely, micelles in the M-PTX-30 group displayed a better distribution, with relatively higher levels in the tumor core, but they also presented with higher variability. **F-G**: For both treatment groups, significantly higher amounts of micelles were found to have extravasated out of the blood vessels, with approx. 80% of the fluorescence signals detected outside of the vessel lumen. Data are presented as average ± SD(n = 4 per group). Unpaired, parametric, two-tailed t-tests were performed to compare the micelles in the core versus periphery of both groups as well as the amount of intravascular vs. extravasated micelles. P values: **** < 0.0001.

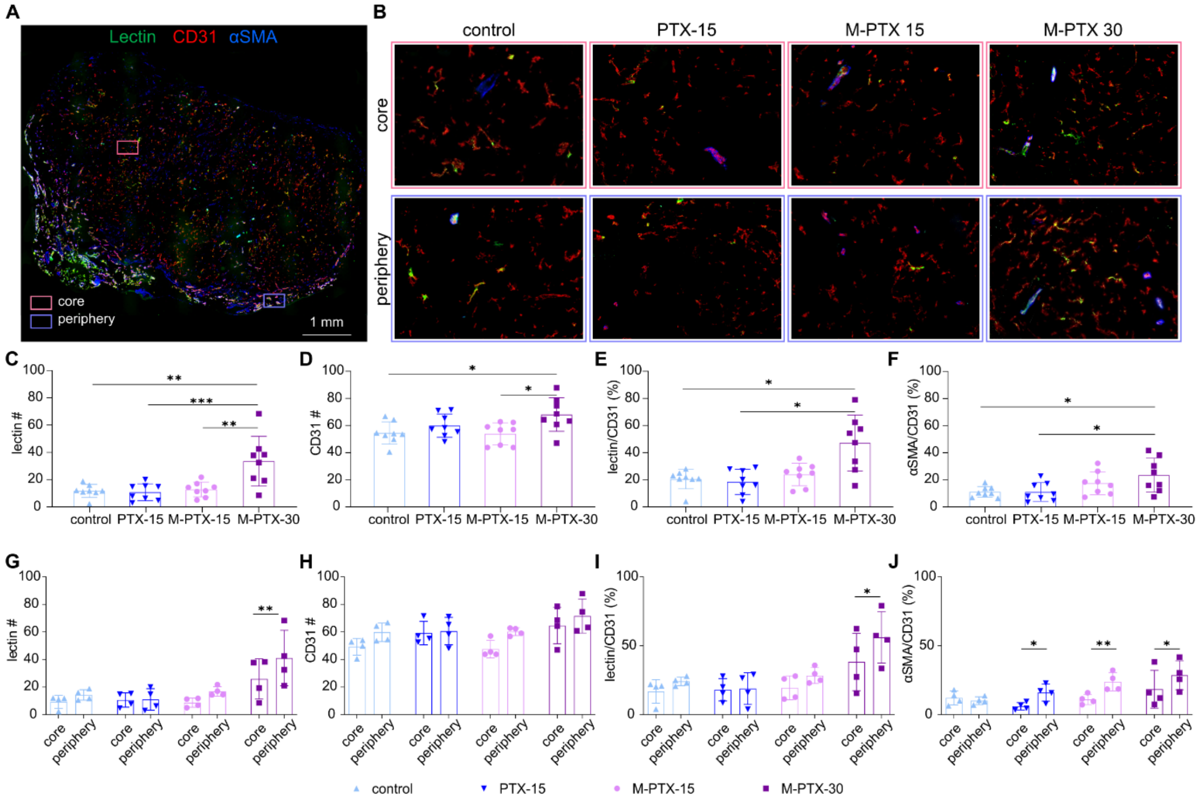

Figure S7: Analysis of tumor vasculature features upon taxane-based (nano)therapy. **A**: Representative whole-tumor fluorescence microscopy image showing the density and distribution of blood vessels (CD31), perfused blood vessels (lectin) and pericyte coverage (αSMA). As in Figures S5 *and* S6, tumors were divided into two regions, considering the inner 75% as the core and the outer 25% as the periphery. **B**: Representative zoom-in images for the 4 treatment groups. **C-F**: Treatment with M-PTX-30 promoted tumor vascularization. Vessel density (**D**), perfusion (**C**), functionality (**E**), and maturation (**F**) were positively affected by double-dose micelle treatment. Data are presented as average ± SD (n = 4 per group). Panels C, D, F: unpaired, parametric one-way ANOVA with Tukey correction was performed to analyze statistical differences between the groups. Panel E: statistical differences were analyzed via unpaired, non-parametric one-way ANOVA with Dunn's correction. P values: * < 0.05, ** < 0.01, *** < 0.001. **G-J**: In terms of differences in tumor vasculature features in core vs. periphery, we observe that particularly for the double-dose micelles treated group, vessel perfusion, functionality and maturity were increased in the periphery vs. in the core of tumors. Data are presented as average ± SD (n = 4 per core, n = 4 per periphery). Statistical differences were analyzed via two-way ANOVA and Bonferroni correction. P values: * < 0.05, ** < 0.01.

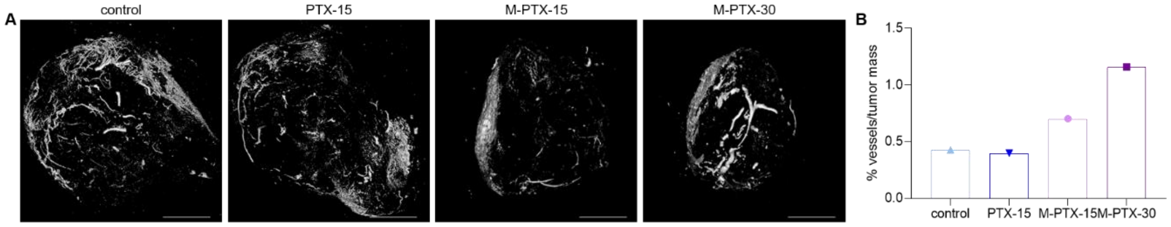

Figure S8: Analysis of tumor blood vessel perfusion using micro-CT. A: One tumor per group was perfused with the vascular casting agent Microfil to visualize and quantify the tumor vascularization. Ex vivo micro-CT imaging indicated that treatment with M-PTX-30 resulted in relatively more and in relatively larger vessels being perfused. **B**: Quantification of the extent of functional tumor vascularization, determined via assessing the percentage of Microfil-positive voxels as part of the whole tumor mass. Scale bar = 2 mm.

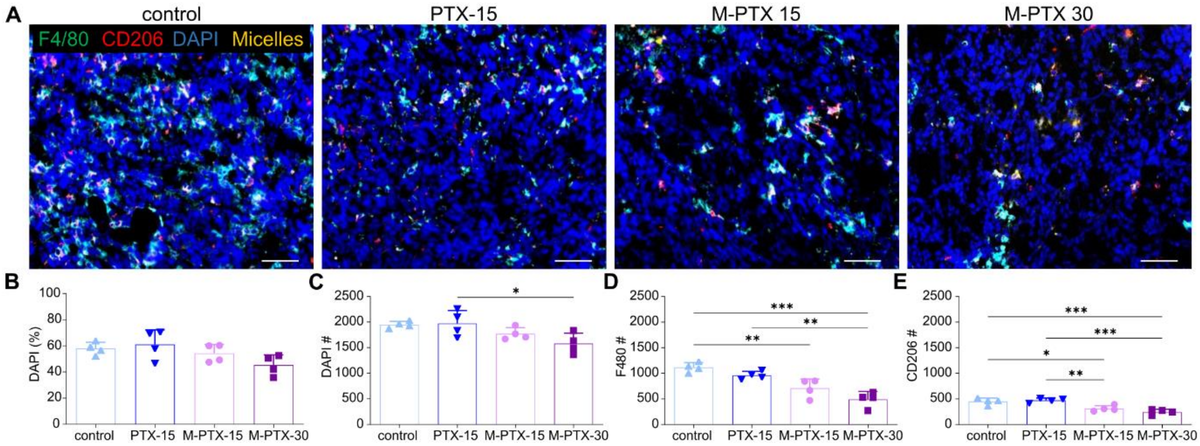

Figure S9: Analysis of cellular and macrophage density in tumors upon taxane-based (nano)therapy. A: Representative fluorescence microscopy images showing DAPI$^+$ cell nuclei, F4/80$^+$ (i.e. all) macrophages and CD206$^+$ (M2-like) macrophages in tumors for the control, PTX-15, M-PTX-15 and M-PTX-30 treatment groups. Scale bar = 60 µm. **B**-**C**: Tumor cellularity was analyzed by quantifying the area fraction of DAPI as well as the number of DAPI-stained nuclei. Cellular density was found to be reduced upon treatment with micellar PTX. **D-E**: The number of F4/80$^+$ and CD206$^+$ macrophages significantly decreased upon treatment with micellar PTX. Data are presented as average ± SD (n = 4 per group). Statistical significance was analyzed via unpaired, parametric one-way ANOVA with Tukey correction. P values: * < 0.05, ** < 0.01, *** < 0.001.

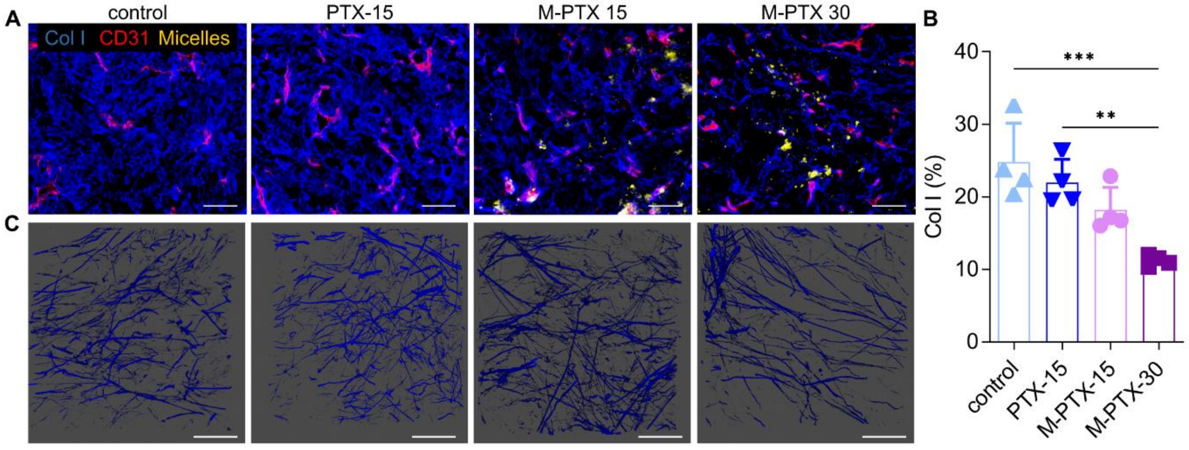

Figure S10: Analysis of collagen density in tumors upon taxane-based (nano)therapy. **A**: Representative fluorescence microscopy images showing collagen I (Col I) and blood vessels (CD31) for control, PTX-15, M-PTX-15 and M-PTX-30. **B**: A dose-dependent decrease of collagen I area fraction was observed upon treatment with M-PTX-30. Scale bar = 60 µm. Data are presented as average ± SD (n = 4 per group). Statistical significance was assessed via unpaired, parametric one-way ANOVA with Tukey correction. P values: ** < 0.01, *** < 0.001. **C**: Representative two-photon laser scanning microscopy images confirming reduced collagen density particularly for M-PTX-30 treatment. Second harmonic generation imaging was employed to visualize collage fibers. Scale bar = 60 µm.

SUPPORTING METHODS

Cy7 polymer conjugation

Cy7 NHS ester was dissolved in dry DMSO, at a concetration of 10 mg/mL. The polymer (31 mg), 1 µL of dry TEA and 0.18 mL of Cy7 NHS ester solution were added into a dried flask and the reaction was performed at 50 ^{o}C for 48 h. Uncoupled Cy7 was removed by dialysis against DMSO. The final Cy7-labeled polymer product was collected after freeze-drying. Cy7 conjugation to the polymer was verified via GPC coupled to UV detection at 700 nm.

Histological analysis

Fluorescence stainings were performed on 8 µm-thick tumor cryosections. The slices were washed with PBS, fixed with methanol 80% and -20°C acetone, and washed again with PBS. Primary antibodies (i.e. anti-ki67 (1:100), anti-casp-3 (1:1000; Abcam, UK), anti-CD31 (1:100; BD Biosciences, USA), anti-collagen I (1:100; Novus Biologicals, USA) and anti-αSMA (1:100; (Progen Biotechnik, Germany)) were diluted in 12% bovine serum albumin (BSA) (PAN Biontech, Germany) and applied to the tumor sections for 1 h. Excess antibody was removed via three consecutive PBS washes, and the slices were subsequetly incubated with the respective secondary antibodies, also diluted in 12% BSA (i.e. Cy3 anti-rabbit (1:500), AMCA anti-rabbit (1:50), Cy3 anti-rat (1:500; Dianova, Germany) and DAPI (1:500; Merck, Germany). Unbound antibodies were removed via PBS washes, slides were mounted with Mowiol 4-88 (Carl-Roth, Germany) and the glass-covered to be stored at 4°C. A different protocol was used for the staining of F4/80 and CD206. Slides were fixed with 10% Neutral Buffered Formalin and washed with TrisNaCl-Tween buffer (TNT), and additionally fixed with 80 % methanol and -20°C-cold acetone, followed by another TNT wash. The samples were incubated with a blocking reagent (from PerkinElmer, USA), subsequently with anti-F4/80 primary antibody (1:500; AbD Serotec, Germany) diluted in the blocking reagent for 1 h, and then with PBS HRP anti-rat secondary antibody (Abcam, United Kingdom) for 45 min. Next, fluorescein TSA dye (1:50; PerkinElmer, USA) diluted in the amplification diluent buffer (from PerkinElmer, USA) was applied for 10 min. The antigen-antibody complex was removed via heating the slides immersed in an AR6 buffer solution in a 98°C water bath. After this, the

slides were washed with distilled water and TNT, and incubated with an anti-CD206 primary antibody (1:100; Acris, Germany) and a corresponding Cy3-labeled anti-rat secondary antibody. Haematoxylin and eosin (H&E) staining was done according to standard procedures, on tumor previously fixed in 4% PFA. The sections were dehydrated with ethanol and xylene to be mounted with Vitro-clud (from R. Langenbrinck GmbH, Germany) prior to glass-coverage and microscopy analysis.

Microscopy

Microscopy images of tumor sections were acquired using an AxioImager M2 microscopy system equipped with an AxioCamMRm Rev.3 camera (Carl Zeiss AG, Jena, Germany). A magnification of 10x was used for acquiring overview images, 20x was used for acquiring images for quatification. The overview images were spatially divided into “core” (the inner 75% of the lesion) and “periphery” (the outer 25%). We always acquired 4 representative images per region and per tumor for quantification. Images in the bright-field channel were acquired for the H&E-stained tumor sections. Two-photon laser scanning microscopy (TPLSM) images to assess collagen content (via second harmonic generation (SHG) imaging) were obtained based on 100 µm-thick, unstained and water-immersed cryosections. TPLSM was performed using the Leica Stellaris 8 Dive Falcon multiphoton microscope (Leica Microsystems, Germany).

Microscopy image analysis

The area fraction (AF %) was quantified for the fluorescence signals associated with the Cy7-labeled micelles, Ki67, Cap-3, DAPI and Collagen I. This was done using the AxioVision SE64 Rel. 4.8 software (Carl Zeiss, Germany). The number of $DAPI^+$ nuclei, and $F4/80^+$ and $CD206^+$ macrophages were obtained using the Inform software (PerkinElmer, USA). The cells were segmented and phenotyped as F4/80 and CD206. The processing of the images produced four categories: single positivity of the cell for F4/80, single positivity for CD206, double positivity for F4/80 and CD206, and double negativity. Cellularity was calculated as the total number of $DAPI^+$ segmented cells. The number of $F4/80^+$ macrophages resulted from the percentage of the sum between F4/80 single positivity and F4/80/CD206 double positivity versus the total number of cells. The number of $CD206^+$ macrophages resulted from the percentage of the sum between CD206 single positivity and F4/80/CD206 double positivity over the total number of cells. The number of $CD31^+$, $lectin^+$ and $αSMA^+$ vessels were counted manually and the functionality and maturity were calculated as percentage of lectin# / CD31# and as percentage of αSMA# / CD31#, respectively. Necrosis was segmented with Imalytics Preclinical 2.0, upon thresholding the images based on the different pixel intensities of the necrotic versus the non-necrotic areas. The extent of necrosis was quantified as the ratio between the segmented necrotic area and the total tumor area. TPLSM images were analyzed using the LAS X 3D software (Leica Microsystems, Germany). All microscopy values are presented as average ± standard deviation (n = 4 per group), and all according statistical analyses were performed using GraphPadPrims 9.

In vivo CT-FMT analysis micelle biodistribution

3D CT-FMT reconstructions were segmented and the fluorescence distribution of Cy7-labeled micelles was quantified, converted into percentage of the injected dose (%ID) and normalized to comparable tissue volumes (in cm^3) for tumor, liver, spleen, kidneys, lungs and heart for accumulation on days 3, 7, 10, 14 and 17 after the initiation of micelle-based nano-taxane therapy. The values for % ID cm^{-3} were averaged per group (n = 5) and plotted as average ± standard deviation for each organ and each time point. Statistically significant differences in the % ID cm^{-3} values for organs at different time points were analyzed via two-way ANOVA with Tukey correction, using GraphPadPrism 9 software.

Ex vivo FRI analysis of micelle tumor targeting

2D fluorescence reflectance images (FRI) at an excitation wavelength of 750 nm were acquired for harvested whole tumors as well as for 100 µm-thick tumor tissue cryosections using the VisEn 2500 FMT device (PerkinElmer, USA). Fluorescence units (f.u.) of whole tumors and tumor sections were obtained from the segmentation of the images using Imalytics Preclinical 2.0. The segmented tumor sections were eroded based on the surface areas of the whole tumor tissue slices, in order to compare micelle localization in the tumor periphery (i.e. the outer 25% surface area) to micelle accumulation in the tumor core (i.e. the inner 75% surface area). Data were presented as average ± standard deviation (n = 4 per group). To assess statistical significance, unpaired, parametric, two-tailed t-tests were performed using the GraphPadPrism 9 software.

Ex vivo micro-CT analysis

To 3D visualize and quantify perfused tumor blood vessels upon taxane-based (nano)therapy, 1 mouse per group was intracardially perfused with MicrofilMV-112 (Flow Tech, USA) prior to sacrificing the mice. After Microfil polymerization, tumors were resected and fixed in 10% formalin/PBS. The SkyScan 1272 micro-CT system (SkyScan, Belgium) was employed to acquire high-resolution ex vivo scans of the fixed tumor tissue specimens. Micro-CT scanning layers were subsequently reconstructed into 3D images via employing Feldkamp-type filtered back-projection. The vessels were segmented in a stack of bi-dimensional images using the Imalytics Preclinical 2.0 software. The extent of functional tumor vascularization was calculated as the percentage of the CT-segmented vessel volume versus the respective CT-segmented total tumor volume.
